# The importance of community health workers as frontline responders during the COVID-19 pandemic, Somalia, 2020–2021

**DOI:** 10.3389/fpubh.2023.1215620

**Published:** 2023-08-17

**Authors:** Lilly M. Nyagah, Sulaiman Bangura, Omar Abdulle Omar, Mary Karanja, Mashrur Ahmed Mirza, Hossain Shajib, Haron Njiru, Kumlachew Mengistu, Sk Md Mamunur Rahman Malik

**Affiliations:** World Health Organization, Mogadishu, Somalia

**Keywords:** COVID-19, community health workers, case notification, Somalia, pandemic

## Abstract

**Introduction:**

We examined the contribution of community health workers as frontline responders for the community-based surveillance in Somalia during the first year of the COVID-19 pandemic for detection of COVID-19 cases and identification of contacts.

**Methods:**

We retrieved COVID-19 surveillance data from 16 March 2020 to 31 March 2021 from the health ministry’s central database. These data were collected through community health workers, health facilities or at the points of entry. We compared the number of suspected COVID-19 cases detected by the three surveillance systems and the proportion that tested positive using the chi-squared test. We used logistic regression analysis to assess association between COVID-19 infection and selected variables.

**Results:**

During the study period, 154,004 suspected cases of COVID-19 were detected and tested, of which 10,182 (6.6%) were positive. Of the notified cases, 32.7% were identified through the community-based surveillance system, 54.0% through the facility-based surveillance system, and 13.2% at points of entry. The positivity rate of cases detected by the community health workers was higher than that among those detected at health facilities (8.6% versus 6.4%; *p* < 0.001). The community health workers also identified more contacts than those identified through the facility-based surveillance (13,279 versus 1,937; *p* < 0.001). The odds of COVID-19 detection generally increased by age. Community-based surveillance and health facility-based surveillance had similar odds of detecting COVID-19 cases compared with the points-of-entry surveillance (aOR: 7.0 (95% CI: 6.4, 7.8) and aOR: 7.5 (95% CI: 6.8, 8.3), respectively).

**Conclusion:**

The community health workers proved their value as first responders to COVID-19. They can be effective in countries with weak health systems for targeted community surveillance in rural and remote areas which are not covered by the facility-based surveillance system.

## Introduction

Similar to many other countries in sub-Saharan Africa, Somalia, with its fragile health system, faced a difficult task in responding effectively to the COVID-19 pandemic in the early phase. In addition, with fewer than one skilled health care worker per 1,000 population in a country of nearly 15 million people (1), essential health services were severely disrupted and remained so even 2 years after the start of the pandemic (2). This, coupled with weak surveillance system and limited diagnostic and testing capacity for COVID-19, made early detection of cases and contact tracing among the population even more challenging (3). In addition, poor health-seeking behavior, fear of stigma, the considerable distance to reach health facilities, and the unavailability or unaffordability of or lack of trust in public health services (4) resulted in very few sick people actively seeking health care and testing for COVID-19 resulting in gross underreporting of reported cases of COVID-19. Between 20 March 2020 and 31 March 2023, Somalia reported a total of 27,334 cases of COVID-19 and 1,361 deaths (5), which is likely an underestimation of the true burden and community spread of the disease in the country.

At the time of the COVID-19 pandemic, the country’s only functioning surveillance system was the Early Warning, Alert and Response Network (EWARN). However, the system had poor population coverage, and suboptimal reporting timeliness and completeness (6, 7). With only 795 of an estimated 1,200 health facilities being included in the network during the pandemic (8). This left a large number of districts without any surveillance coverage.

To bridge this gap in surveillance, the World Health Organization (WHO) recruited 3,227 community health workers in 49 priority districts across the country (9). This number was later reduced to 1770 covering 51 districts in Banadir region and Hirsabelle, jubaland, SouthWest, Somaliland, Galmudug and Puntland states of Somalia These CHWs were, in part, recruited as a means of establishing a community-based surveillance system to complement the facility-based EWARN surveillance (9). They were selected from and deployed to the same communities where they lived and had some experience in surveillance as they had previously been recruited for polio surveillance. The main role of these community health workers was to make house-to-house visits and look for any suspected COVID-19 cases using a syndromic-based case definition. They also conducted community engagement and health awareness sessions on simple hygiene measures to reduce the risk of exposure and infection in the community. Epidemiologists from WHO and the Federal Ministry of Health trained the health workers on the case definition, what to do when they identified a suspected case, and basic infection prevention and control measure for themselves, the suspected cases and their close family members (see Supplementary material for more information on the work of the community health workers). Further, the community health workers (CHWs) were trained on ethics and maintaining confidentiality of the communities they served. The CHWs were supervised by a team of rapid responders drawn from among district medical officers, district polio officers and district social mobilization officers. A total of 850 such teams were also deployed in the 49 priority districts. The aim of this complementary community-based surveillance system was to identify cases and contacts early to stop onward transmission and limit sustained community spread of COVID-19 in places which were not covered by the EWARN.

The involvement of CHWs for active case searching and contact tracing is not new. During the Ebola virus disease outbreaks in Africa in 2014–2015 and some other infectious disease outbreaks, such as measles and cholera, community-based surveillance was established in Africa using community health workers, village health volunteers or other Red Cross and Red Crescent volunteers (10, 11). This strategy was effective in stopping the chain of transmission, reducing stigma and improving health-seeking behavior during the acute phase of these epidemics.

The extent to which strategies such as the use of CHWs and community-based surveillance as a complement to overall surveillance have been effective in responding to the COVID-19 pandemic in fragile and humanitarian crisis setting has not been well documented. However, their value and critical importance as first responders to infectious diseases outbreaks and in improving health outcomes in the population they serve are well documented (11–14).

In this study, we examined the role of these community health workers and the effectiveness of community-based surveillance for COVID-19 case identification and contact tracing in Somalia. A similar case-reporting system was used at the points of entry for returning travelers during the initial phase of the pandemic.

## Methods

### Setting and population

We performed a retrospective descriptive analysis of data on all suspected, laboratory-confirmed and recovered cases of COVID-19 and close contacts of laboratory-confirmed cases collected by the community health workers in the 49 priority districts of Somalia between 2020 and 2021. Data from the community-based surveillance were collected and sent to the central database every day using the Open Data Kit (ODK) tool available at https://opendatakit.org. Community based surveillance includes active detection of suspected cases of diseases under surveillance as well as other surveillance activities such as detection of outbreak alerts and rumors for verification purpose which are conducted at the community level primarily through house-to-house visits while facility-based surveillance means passive detection of suspected cases or diseases of outbreak potentials in the health-facilities with or without appropriate follow-up of clinical progression of diseases in the communities. The districts consisted of both rural and urban areas and represented 62% of the population of Somalia. Most of these districts are in hard-to-reach areas because of insecurity and other access constraints such as poor road infrastructure and long distances between the settlements and the nearest health facility. Data from the EWARN on all suspected cases detected passively in health facilities and their laboratory-confirmation status and outcome were also collected daily. Similarly, data on laboratory-confirmed cases of COVID-19 and suspected cases detected at the points of entry in returning travelers were also collected daily.

At the time of COVID-19 pandemic, the country’s only functioning surveillance system was EWARN which was established in 2017 to detect only 15 epidemic-prone diseases using a syndromic-based case definition (6). The EWARN system in Somalia is used for reporting of diseases and events in the community and at the facilities. However, for ports of entry, there is no system for disease and events reporting. Until now, the country does not have any other functioning and comprehensive disease surveillance system such as the integrated disease surveillance and response system (IDSRS) like many African countries.

### Data collection

We extracted data from the central database of cases of COVID-19 hosted by the Government of Somalia in its national public health laboratory. Data from the electronic database of the EWARN on the number of suspected and laboratory-confirmed cases of COVID-19 identified in the health facilities were sent automatically every day to this central database. In addition, data from the community-based surveillance on the number of suspected, tested and laboratory-confirmed cases detected in the community were also sent every day to this central database using the ODK tool. An electronic line list was developed to facilitate the community health workers in contact listing, tracing and follow-up. The ODK tool was also used to collect and send surveillance data on suspected and laboratory-confirmed cases of COVID-19 at points of entry. Data collected from each of these three surveillance systems included sociodemographic variables such sex, age, residence (urban, rural and camp for internally displaced people), state of residence and basic clinical presentation at notification. The central database was linked to laboratory test results making it easy to identify and disaggregate cases by case classification and sociodemographic characteristics.

### Data management and analyzes

All COVID-19 case data collected from March 2020 to March 2021 were downloaded from the central database. These data were merged to create a country line-list and were analyzed using Stata statistical software (version 17). We report frequencies and proportions by selected individual characteristics and reporting sources. We tested for the independence of proportions using the Pearson chi-squared test and compared medians using the Wilcoxon rank-sum test. We used bivariate and multivariable logistic regression analysis to assess factors associated with the detection of COVID-19. Factors associated with COVID-19 outcomes that were significant in the bivariate analysis (*p* < 0.05) were included in the multivariable model.

### Ethical considerations

We used routine surveillance data with no personal identifiers in our analyzes. The study was approved by the Federal Ministry of Health, Somalia. Furthermore, the community health workers are trained to maintain a high level of confidentiality.

## Results

### Case notification

**Figure 1:**
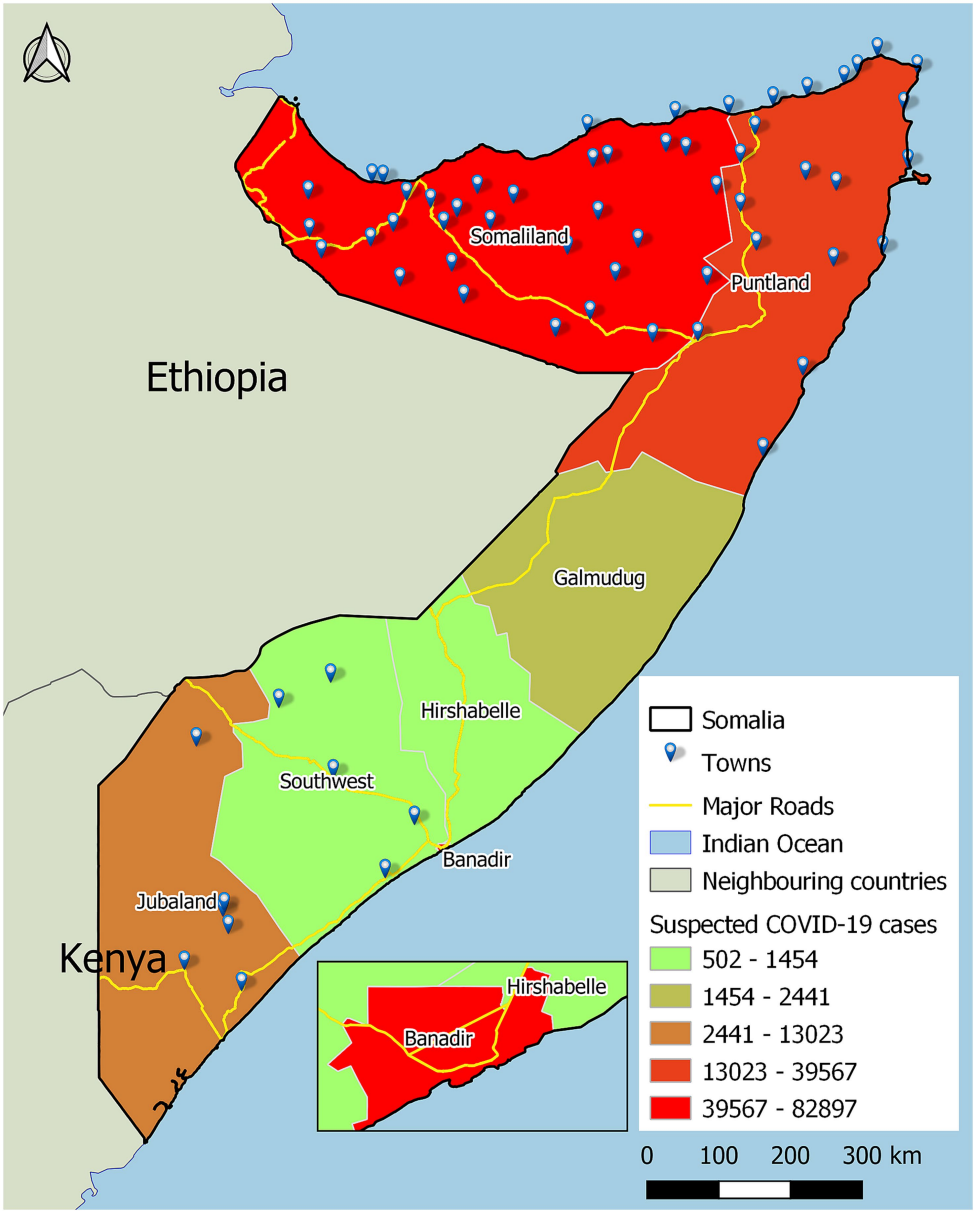
Map of Somalia with suspected COVID-19 cases by state, and display of locations of towns and inset, the Banadir zone, where the capital Mogadishu is situated.

From 16 March 2020 to 31 March 2021, 154,004 suspected COVID-19 cases were tested for SARS-CoV-2, 10,182 (6.6%) of whom were positive. Most COVID-19 suspected cases were reported in states which had a higher concentration of towns, and in the Banadir region, where the Somalia capital Mogadishu is situated (Table 1 and Figure 1). The proportion of males infected with SARS-CoV-2 was higher than females, 6.7% versus 6.4% (*p* = 0.048). SARS-CoV-2 infection was highest in persons 50 years and older (*p* < 0.001). A significantly greater proportion of people living in rural areas (21.3%) were positive for COVID-19 than those living in urban areas (6.5%) and camps for internally displaced people (8.6%) (p < 0.001).

**Table 1:** COVID-19 cases by sociodemographic characteristics and surveillance system, Somalia, 16 March 2020 to 31 March 2021.

Characteristic	Total tested^a^	Positive^b^	Negative^b^	*p*-value^c^
	*n* (%)	*n* (%)	*n* (%)	
Total	154,004 (100.0)	10,182 (6.6)	14,3,822 (93.4)	
Sex				0.048
Female	49,760 (32.3)	3,199 (6.4)	46,561 (93.6)	
Male	104,235 (67.7)	6,980 (6.7)	97,255 (93.3)	
Age group, in years				<0.001
0–9	5,599 (3.6)	78 (1.4)	5,521 (98.6)	
10–19	11,067 (7.2)	488 (4.4)	10,579 (95.6)	
20–29	41,429 (26.9)	2,909 (7.0)	38,520 (93.0)	
30–39	38,705 (25.1)	2,531 (6.5)	36,174 (93.5)	
40–49	23,688 (15.4)	1,294 (5.5)	22,394 (94.5)	
≥50	33,261 (21.6)	2,868 (8.6)	30,393 (91.4)	
Residence				<0.001
IDP	58 (0.0)	5 (8.6)	53 (91.4)	
Rural	694 (0.5)	148 (21.3)	546 (78.7)	
Urban	15,3,241 (99.5)	10,029 (6.5)	14,3,212 (93.5)	
Surveillance system				<0.001
Community-based	50,449 (32.8)	4,349 (8.6)	46,100 (91.4)	
Health facility (EWARN)	83,230 (54.0)	5,338 (6.4)	77,892 (93.6)	
Points of entry	20,325 (13.2)	495 (2.4)	19,830 (97.6)	

a Column percentage.

b Row percentage.

c Chi-squared test.

The community health workers, through active case search, detected 32.7% of all the suspected cases of COVID-19 reported in the country from 16 March 2020 to 31 March 2021, EWARN, based on people attending health facilities, detected 54.0 and 13.2% were detected among returning travelers at points of entry.

Of the 50,449 suspected COVID-19 cases detected through by the community health workers, 8.6% tested positive for COVID-19, while in the facility-based EWARN system, 6.4% of suspected cases tested positive for COVID-19, and in the points-of-entry surveillance, 2.4% tested positive (*p* < 0.001).

In Jubaland, Southwest state and Somaliland, a greater proportion of positive cases were detected by the CHWs (99.5, 64.7 and 58.6%, respectively) compared to health facilities (0.5, 35 and 34.5%, respectively) (*p* < 0.001) (Table 2).

**Table 2:** Total suspected and positive cases of COVID-19 by state and proportion positive by state and surveillance system, Somalia, 16 March 2020 to 31 March 2021.

State	Total number of suspected cases	Positive cases			
		Total	Community-based surveillance	Health-facility based surveillance	Points-of-entry surveillance
		*n* (% of suspected cases)	*n* (%)^a^	*n* (%)^a^	*n* (%)^a^
*Total*	154,004	10,182 (6.6)	4,349 (42.7)	5,338 (52.4)	495 (4.9)
Banadir	82,897	1,945 (2.3)	798 (41.0)	1,136 (58.4)	11 (0.6)
Galmudug	2,233	535 (24.0)	201 (37.6)	334 (62.4)	0 (0.0)
Hirshabelle	502	180 (35.9)	64 (35.6)	116 (64.4)	0 (0.0)
Jubaland	2,752	845 (30.7)	841 (99.5)	4 (0.5)	0 (0.0)
Puntland	19,870	2,872 (14.5)	193 (6.7)	2,435 (84.8)	244 (8.5)
Somaliland	44,491	3,448 (7.7)	2,021 (58.6)	1,188 (34.5)	239 (6.9)
Southwest	1,259	357 (28.4)	231 (64.7)	125 (35.0)	1 (0.3)

a Row percentage of total positive cases.

Comparison of proportion of positive cases by state and surveillance system, *p* < 0.001.

### Identification of contacts

The CHWs identified and traced more close contacts per laboratory-confirmed case than those detected at the health facilities: 13,279 versus 1,937; *p* < 0.001 (Table 3). The number of close contacts identified through the community-based surveillance system was more than double the number identified by the facility-based EWARN system (7 versus 3; *p* < 0.001). The median number (interquartile range (IQR)) of close contacts per laboratory-confirmed case identified and tracked by the community health workers was 4 (IQR 2–6) compared with 3 contacts (IQR 2–4) at the health facility level (p < 0.001). No contacts were identified through the points-of-entry surveillance.

**Table 3:** Close contacts identified for laboratory-confirmed cases of COVID-19 by surveillance system, Somalia, 16 March 2020 to 31 March 2021.

Surveillance system	Number of laboratory-confirmed cases	Number of contacts identified	Number of close contacts identified for each case	Median number of contacts identified (IQR)
Community-based	50,449	13,279	7	4 (2–6)
Facility-based	83,230	1,937	3	3 (2–4)
Points of entry	20,325	0	–	–

IQR, interquartile range.

*p* < 0.001 (Wilcoxon rank-sum test).

### Factors associated with case notification

The likelihood of COVID-19 detection generally increased by age, except for persons aged 40–49 years. Individuals living in camps for internally displaced people had lower odds of having COVID-19 (aOR: 0.3; 95% CI: 0.1, 0.7) compared with urban residents. All regions had higher odds of detection of COVID-19 compared with Banadir; Hirshabelle had the highest likelihood of detection of COVID-19 (aOR: 23.5; 95% CI: 19.4, 28.4). The odds of detection of COVID-19 cases by the community-based surveillance system were similar to those of the facility-based EWARN system compared with the points-of-entry surveillance: aOR: 7.0; 95% CI: 6.4, 7.8 and aOR: 7.5; 95% CI: 6.8, 8.3, respectively (Table 4).

**Table 4:** Multivariable logistic regression analysis of factors associated with detection of COVID-19 cases, Somalia, 16 March 2020 to 31 March 2021.

Variable	Crude OR (95% CI)	Adjusted OR (95% CI)
*Sex*		
Female	ref.	ref.
Male	1.0 (1.0,1.1)	1.0 (0.9, 1.0)
*Age group, in years*		
0–9	ref.	ref.
10–19	3.3 (2.6,4.2)	2.7 (2.1, 3.4)
20–29	5.3 (4.3,6.7)	4.1 (3.3, 5.2)
30–39	5 (3.9,6.2)	4.0 (3.2, 5.1)
40–49	4.1 (3.2,5.1)	3.6 (2.8, 4.5)
≥50	6.7 (5.3,8.4)	5.6 (4.5, 7.1)
*Type of residence*		
Urban	ref.	ref.
IDP	1.3 (0.5,3.4)	0.3 (0.1, 0.7)
Rural	3.9 (3.2,4.6)	1.0 (0.8, 1.2)
*State*		
Banadir	ref.	ref.
Galmudug	13.1 (11.8,14.6)	13.1 (11.8, 14.6)
Hirshabelle	23.3 (19.3,28.1)	23.5 (19.4, 28.4)
Jubaland	18.4 (16.8,20.2)	18.8 (17.0, 20.8)
Puntland	7 (6.6,7.5)	12.0 (11.3, 12.8)
Somaliland	3.5 (3.3,3.7)	4.6 (4.3, 4.9)
Southwest	16.5 (14.5,18.8)	17.3 (15.1, 19.7)
*Surveillance system*		
Points of entry	ref.	ref.
Community-based	3.8 (3.4,4.2)	7.0 (6.4, 7.8)
Facility-based	2.7 (2.5,3)	7.5 (6.8, 8.3)

OR, odds ratio; CI, confidence intervals; ref., reference category; IDP, camp for internally displaced people.

## Discussion

Somalia’s health system has been severely weakened and fragmented by underinvestment resulting from prolonged war, political instability and ongoing humanitarian crisis. As a result of insecurity and poor road infrastructure, many communities are unable to access health and other essential services (15).

In this study, we examined the importance of community-based surveillance systems and CHWs as frontline responders to COVID-19. Our results show that the community-based surveillance reported a third of all suspected cases detected in the country during the first year of the COVID-19 pandemic in Somalia, while about half of the cases were detected at health facilities. The positivity rate of cases identified by the CHWs was higher than that of health facilities or at points of entry indicating its higher sensitivity compared to that of health-facility surveillance. It also supports the usefulness of applying a broader syndromic case definition for early detection of cases during the community spread of COVID-19.

Importantly, the CHWs identified more close contacts of COVID-19 cases than those identified for COVID-19 cases detected at health facilities or points of entry. This is likely because EWARN relied on passive reporting from health facilities and it lacked an adequate number of field workers for optimal contact tracing.

Our study confirms that community-based surveillance can be effective in countries with weak and fragile health systems for targeted community surveillance in rural and remote areas with low coverage of facility-based surveillance system. The value of community-based surveillance to complement existing facility-based surveillance has been reported in other settings and in response to other infectious diseases such as measles, cholera and hepatitis E in Africa as well as for high-threat pathogens such as the Ebola virus (10, 11). In humanitarian crises, health services are often disrupted and detection and testing services for infectious diseases are largely inaccessible because of security and other operational constraints. In these settings, considerable evidence is available on the important role the CHWs play in increasing access to health services for populations in the remote and underserved settings and containing the spread of infection (16). For example, in Somalia, the village polio volunteers program was successful in containing the polio virus outbreak in 2014 and maintaining Somalia’s wild polio virus-free status (17). Similar success in using community-based surveillance has been reported in Niger and the United Republic of Tanzania (18, 19).

Our findings concur with those seen in other African settings such as Niger where community-based surveillance during the COVID-19 pandemic resulted in increased community awareness, detection and notification of COVID-19 cases, and subsequent identification of contacts of confirmed cases (20). Although our study findings are not comparable with high-or middle-income countries which have strong health systems, in Thailand, which also had an acute shortage of health professionals in rural areas, the mobilization of village health volunteers as part of community-based surveillance of COVID-19 resulted in containment of the virus without the need for country-wide lockdown or widespread testing (21).

Compared with points-of-entry surveillance, our results show that community-based surveillance had seven times higher odds (aOR: 7.0; 95% CI: 6.4, 7.8) of detection of COVID-19 cases, and similar to facility-based surveillance (aOR: 7.5; 95% CI: 6.8, 8.3). These data demonstrate that the CHWs can complement the public health workforce in countries where there are shortages of health workers. In addition, if trained and well equipped, they can be first responders in disease outbreaks for identifying potential cases and contacts. The data also highlight the importance of the facility-based EWARN in Somalia and demonstrate its ability to rapidly detect cases following scale up to include COVID-19 in its surveillance and reporting system (22).

The CHWs also performed other functions such as raising community awareness of disease risk factors and symptoms, promoting appropriate preventive practices at the household level, tackling stigma and disseminating messages on health hygiene (9). When factors such as distance, lack of transport, financial issues or other health system barriers preclude the community from accessing health services, informal health care providers can fill the gap. For example, during the Ebola virus outbreak in 2014, the CHWs were part of outbreak management and were involved in social mobilization, house-to-house visits, community meetings, active case finding and contact tracing (10, 11, 23). Although evidence demonstrates the value of the CHWs in improving the health of populations in low and middle-income countries (13, 24), our study also showed their value as contact tracers and case detection through active case search using a syndromic case definition. Other studies have reported that countries with well-established community health worker programs have used them as contact tracers and to raise awareness in the community (25). Indeed, community health workers served as first responders to COVID-19 in high-income countries (26–28).

Our study had some limitations. Data were incomplete for some of the variables. We were also not able to obtain all the data on clinical outcome of all cases and contacts identified though the three surveillance systems, or the effectiveness of the CHWs in interrupting the chain of transmission. Such information would have been useful in understanding the relative value of CHWs for effective case follow-up and their ability to apply public health measures that are essential for stopping community spread. In addition, we could not determine the risk of exposure to the CHWs themselves. However, anecdotal data suggest that this may have been minimal as no absenteeism was reported. Despite these limitations, we believe our study demonstrates the crucial role that the CHWs can play for early disease detection during a pandemic such as COVID-19. However, widespread lockdowns and reluctance of clients to visit healthcare facilities were widely reported in many settings at the onset of the COVID-19 pandemic and could have resulted in higher detection rates of these cases at the community level by the CHWs. The cultural and societal norms of the region studied, familiarity and proximity of the CHWs to the study participants may have also helped in the success recorded when compared to point of entry surveillance and health facility-based surveillance. A strength of our study is that all data on case identification and contact notification reported from the communities, at health facilities and at points of entry either actively or passively were included. This shows that these three surveillance systems can play complementary roles in outbreak response in countries with fragile health systems.

The results of our study support greater investment in the work of CHWs. These health workers have traditionally been used to extend health services at the community level, particularly in underserved or remote populations (29), but they are also a vital part of the outbreak and pandemic response to prevent and reduce the spread of infection. We therefore recommend continuous capacity-building of the CHWs to enhance their skills to manage the evolving health needs of the community. By virtue of their understanding of the local context and the trust the people they serve have in them, they can be a crucial link between the community and the health system in fragile settings.

## Data availability statement

The raw data supporting the conclusions of this article will be made available by the authors, without undue reservation.

## Ethics statement

The studies involving humans were approved by Federal Ministry of Health, Somalia. The studies were conducted in accordance with the local legislation and institutional requirements. Written informed consent for participation was not required from the participants or the participants' legal guardians/next of kin in accordance with the national legislation and institutional requirements.

## Author contributions

LN, SB, OO, MK, MM, HS, HN, KM and SM contributed to conception and design of the study. OO organized and maintained the database. SM provided guidance and support throughout the research process and analysis and wrote sections of the manuscript and critically reviewed the whole paper. LN undertook the statistical analysis and wrote the first draft of the manuscript. All authors contributed to the article and approved the submitted version.

## Funding

The WHO country office received funding from the Foreign, Commonwealth & Development Office of the United Kingdom of Great Britain and Northern Ireland (OCR S FCDO-SOM-74493), Government of Germany (OCR S DE COVID 2022 PILLAR10Vx-73579), Global Fund (OCR S UNICEF C19RM-72947), GAVI (OCR S GAVI COVID19 VACCINE-72300) and the World Bank to strengthen community-based surveillance system in the country (OCR S DE COVID-19 SPRP 2022-71400), Global Affairs Canada (OCR S DFATD ACT-A HSC EMRO-71862).

## Conflict of interest

The authors declare that the research was conducted in the absence of any commercial or financial relationships that could be construed as a potential conflict of interest.

The handling editor OO declared a shared parent affiliation with the authors at the time of review.

## Publisher’s note

All claims expressed in this article are solely those of the authors and do not necessarily represent those of their affiliated organizations, or those of the publisher, the editors and the reviewers. Any product that may be evaluated in this article, or claim that may be made by its manufacturer, is not guaranteed or endorsed by the publisher.
