# Supplementary material for: The importance of community health workers as frontline responders during the COVID-19 pandemic, Somalia, 2020–2021
Source: Front Public Health. 2023 Aug 17;11:1215620. doi: 10.3389/fpubh.2023.1215620 (PMC10469613; doi:10.3389/fpubh.2023.1215620)
Supplement: Supplementary file 1 [file Table_1.DOCX]

**Box 1: Key activities of community health workers in responding to COVID-19**

- Educating the communities they engage with about the signs, symptoms and transmission routes of COVID-19. This learning could also include building skills among the community on personal preventive measures, such as wearing a mask, maintaining physical distancing, hand hygiene, coughing into elbows, and water, sanitation and hygiene (WASH) interventions.
- Mobilizing local residents to use hand-washing stations in the communities and health facilities.
- Identifying the signs and symptoms of COVID-19 in community members and reporting suspected cases immediately to district polio officers and district medical officers.
- Communicating daily health information validated by WHO to the community and combatting the spread of misinformation, rumours and fears.
- Following up with, monitoring and supporting patients who are self-isolating or in quarantine in the community and ensuring delivery of food and social and medical support through the local leaders.
- Monitoring COVID-19 patients for clinical deterioration, reporting patients with worsening illness to the district medical officer and supporting the rapid referral of individuals who require hospitalization, thus reinforcing links between the health system and communities.
- Undertaking contact tracing and symptom scoring, and monitoring contacts of patients with COVID-19 and reporting immediately to the district response team if anyone develops signs and symptoms of COVID-19.
- Supporting the WASH teams to identify homes and high-risk surfaces in communities for disinfection.
- Promoting the use of essential health services, e.g. encouraging mothers to take children for routine immunization and referring children with dangerous signs of dehydration to health facilities.

Household or community member alerts the CHWs in a district when a person has COVID-19 symptoms.

Patient has complications, e.g. difficulties breathing, or has comorbidities.

Patient has symptoms, e.g. fever and cough, but is able to stay at home.

RRT explains to the patient and family the need for the patient to self-isolate.

The RRT advises the patient to self-isolate and traces all contacts to inform them of his/her condition and encourage them to be aware and get tested.

If results are positive, the RRT advises the patient to isolate and refers him/her to an isolation centre for medical support.

Patients are looked after and discharged only after they test negative for COVID-19.

A person is asymptomatic, i.e. has tested positive for COVID-19, but has no symptoms.

CHW asks him/her to isolate at home, and to avoid contact with anyone else for at least 2 weeks.

A person is symptomatic, i.e. has COVID-19 and has symptoms, the CHWs alerts district RRT.

The district RRT visits the household to verify the case and collect samples for testing. Test samples are sent to the nearest laboratory. The patient is advised to self-isolate until results are known.

CHW checks on people in isolation for 14 days, after which they are retested for COVID-19.

CHW informs them they have been in contact with a suspected or confirmed case of COVID-19.

CHW traces all people the patient has had contact with in the past 14 days.

Community health care workers visit households in their area as part of their routine search for COVID-19 cases. They ask families to report if they have (or anyone they know has) symptoms. Using the case definition for COVID-19 as guidance, they determine whether there are any suspected cases.

RRT follows up with patient to see how he/she is doing for 14 days during their regular household visits.

CHWs wear face masks and restock soap/antibacterial agents for hand hygiene every day. They have microplans/maps ready for visits.

**Figure 1: Sequence of actions when community health workers meet cases of COVID-19**

CHW: community health worker; RRT: rapid response team.
